# Epithelium Expressing the E7 Oncoprotein of HPV16 Attracts Immune-Modulatory Dendritic Cells to the Skin and Suppresses Their Antigen-Processing Capacity

**DOI:** 10.1371/journal.pone.0152886

**Published:** 2016-03-31

**Authors:** Janin Chandra, Yan Miao, Natasha Romoff, Ian H. Frazer

**Affiliations:** University of Queensland Diamantina Institute, Translational Research Institute, Woolloongabba, Queensland 4102, Australia; University of Tokyo, JAPAN

## Abstract

Antigen presenting cells (APCs) in skin can promote either antigen-specific effector functions or antigen tolerance, and thus determine clearance or persistence of cutaneous viral infections. Human papillomavirus (HPV) infections can persist in squamous epithelium in immunocompetent individuals, and some persisting HPV infections, particularly with HPV16, promote malignant epithelial transformation. Here, we investigate whether local expression of the HPV16 protein most associated with malignant transformation, HPV16-E7, affects the phenotype and function of APC subsets in the skin. We demonstrate an expanded population of Langerhans cells in HPV16-E7 transgenic skin with distinct cell surface markers which express immune-modulatory enzymes and cytokines not expressed by cells from non transgenic skin. Furthermore, HPV16-E7 transgene expression in keratinocytes attracts new APC subsets to the epidermis. In vivo migration and transport of antigen to the draining lymph node by these APCs is markedly enhanced in HPV16-E7 expressing skin, whereas antigen-processing, as measured by proteolytic cleavage of DQ-OVA and activation of T cells in vivo by APCs, is significantly impaired. These data suggest that local expression of HPV16-E7 in keratinocytes can contribute to persisting infection with this oncogenic virus, by altering the phenotype and function of local APCs.

## Introduction

Infection of the anogenital epithelium with an oncogenic human papillomavirus (HPV) initiates 99% of cervical cancers in women. While 98% of infections with HPV16, the genotype most commonly associated with cervical cancer, will be cleared within 5 years, the immune response responsible for eliminating infection is slow, and prolonged viral persistence is associated with increasing risk of cancer [1]. A variety of studies suggest that increased regulatory T cells in lesions correlate with virus persistence and cancer progression, while regressing lesions show a dominance of CD8^+^ T cell infiltrates [2–4].

Amongst myeloid cells with antigen presenting capacity, conventional dendritic cells (cDCs) can control immune tolerance and immunity by enabling maturation of naïve T cells to a regulatory or cytotoxic phenotype [5]. cDCs can be distinguished by their specific location in organs and tissues. Some reside in secondary lymphoid tissues, where they receive antigens and danger signals either via blood or lymph, while others are located in non-lymphoid tissues such as the lung or mucosal surfaces, where they are directly exposed to pathogens. These latter cDCs can migrate to tissue-draining lymph nodes, and either transfer antigens to lymph node-resident cDCs or themselves initiate T cell responses. Lymph nodes host both resident and migratory cDC subsets. In steady state in mice, two main groups of cDCs can be found, distinguished by their differential expression of CD11b [6]. CD11b^+^ DCs include lymph node-resident CD4^+^CD11b^+^ or CD4^-^CD8^-^CD11b^+^ DCs and also non-lymphoid tissue CD11b^+^ DCs including classical dermal CD11b^+^ DCs and CD207^+^Epcam+ Langerhans cells (LCs). CD11b^+^ DCs are specialized in the activation of CD4^+^ T helper cell responses [7–9]. CD11b^-^ DCs consist of the lymph node-resident CD8^+^ DCs and the dermal CD207^+^CD103^+^ DCs. Both DCs are ontogenetically related and share common functions such as the ability to cross-present antigen and the activation of CD8^+^ T cells [7, 10].

The skin represents the first barrier of defence against pathogens from the outside world [11]. While ensuring that harmful microbes are recognized and defended, the skin also ensures that beneficial microbiota living on the skin are tolerated. Skin-resident DCs play a major role in balancing these processes. LCs are a unique set of self-renewable DCs of the epidermis that account for 5% of the total nucleated epidermal cells [12], whereas classical dermal CD11b^+^ and CD207^+^CD103^+^ DCs are found in the dermis. All skin-resident DCs have the ability to migrate to the skin-draining lymph node to modulate adaptive cell-mediated immunity. In steady state, non-lymphoid dermal CD103^+^ DCs in mice and CD141^+^ DCs in human maintain tolerance in the skin through the induction of regulatory T cells [13, 14]. However, during skin infection, CD103^+^ DCs can activate cognate effector T cells either directly or transfer antigen to lymph node-resident CD8^+^ DCs [7, 15, 16]. Clearance of viral infections in the skin, e.g. herpes simplex virus, depends on these processes [7].

Because HPV clearance is associated with a CD8^+^ T cell response which is primed by cDCs, we analysed the phenotype of skin-resident DCs in K14.E7 mice, in which the expression of the non-structural oncoprotein HPV16-E7 as a transgene is driven by the keratinocyte promoter K14 (K14.E7) in murine skin and leads to hyperplasia in the epidermis [17]. Skin grafts from K14.E7 transgenic mice are not rejected by immune competent non-transgenic recipients, despite the expression of HPV16-E7 as a non-self antigen [18]. We have previously shown that a variety of immune factors in HPV16-E7-expressing skin including NKT cells, IFNγ, IL-17 and mast cells generate immune tolerance blocking graft rejection [19–21].

We have recently shown that LC homeostasis and antigen-uptake is altered in K14.E7 skin [22]. Following up on this previous study, we here report that all DC numbers are elevated in K14.E7 skin, and that MHCII, CD11b and CD207 (Langerin) expression is reduced in K14.E7 LCs. K14.E7 epidermal LCs further express a variety of immune-modulatory enzymes and cytokines. All DCs subsets from K14.E7 mice are competent to take up antigens in the skin and migrate to the lymph nodes. When isolated from skin, un-manipulated K14.E7 skin-derived DCs are also able to induce CD8^+^ T cell proliferation in vitro. However, within the K14.E7 skin specific immunological microenvironment, skin-resident K14.E7 DCs have impaired ability to process antigen and prime CD8+ antigen-specific T cells, suggesting that the immune-suppressive HPV-E7-expressing skin effects DC functionality.

## Results

### Characterising APCs in the epidermis of K14.E7 mice

Following skin infection, APCs residing in the skin are generally activated by danger- and pathogen-associated molecular patterns (DAMPs and PAMPs) from dying cells, and by induced immunoregulatory cytokines. Infection of epithelial keratinocytes with HPVs is however persistent and non-lytic, resulting in epithelial hyperproliferation without evident local inflammation. To examine presentation of pathogen-derived antigen under these circumstances, we used a mouse in which the HPV16 E7 oncoprotein is expressed from a keratin promoter with resultant epithelial hyperplasia (K14.E7 mice). The epithelial hyperplasia observed in K14.E7 mice is particularly dominant in the ear skin and is associated with infiltration of a variety of immune cells into the skin including myeloid cells, thus serving as a model to study immune regulation in HPV-associated pre-malignant skin lesions [19, 23, 24]. However, the composition of myeloid cells and in particular the function of local APCs remains undetermined.

**Fig 1:**
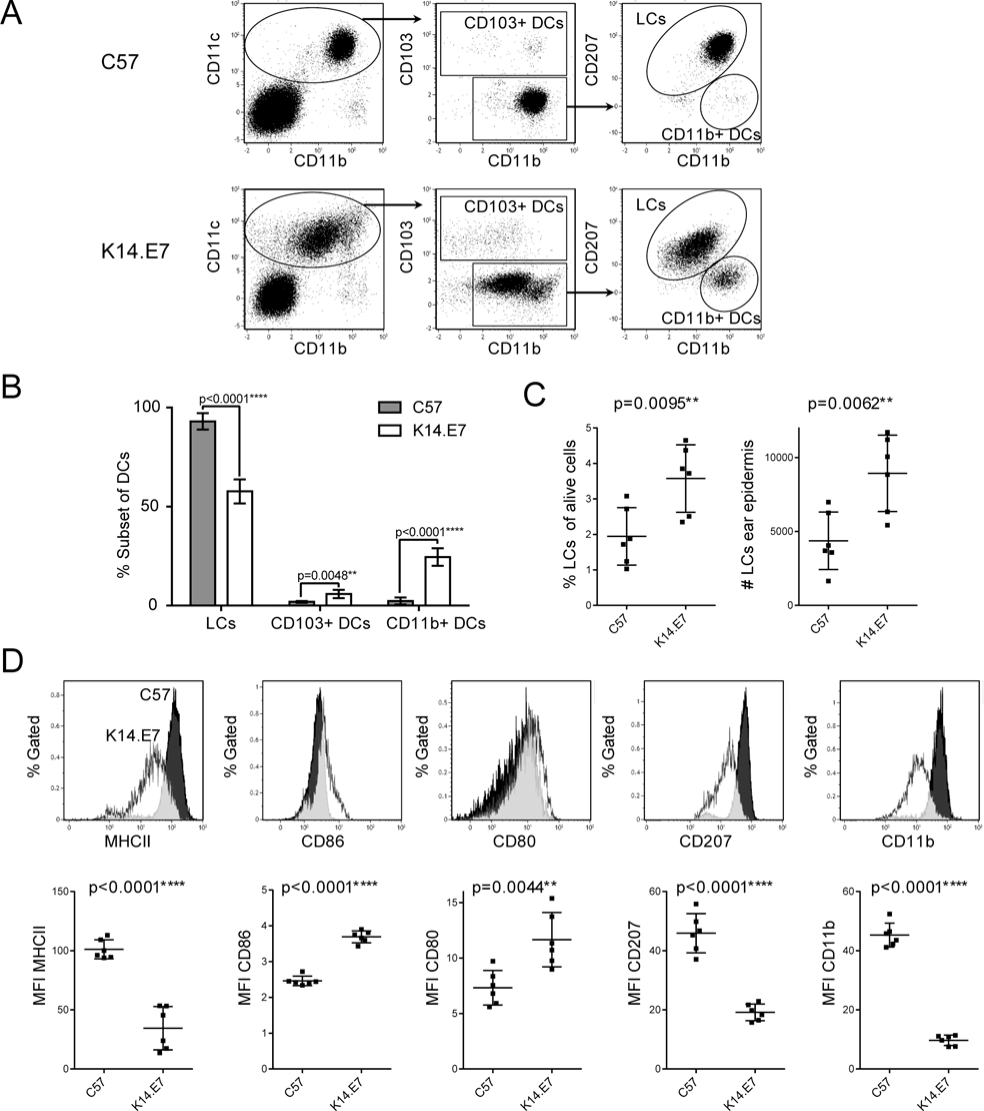
Antigen presenting cells are increased and of altered phenotype in K14.E7 transgenic mouse epidermis. Single cell suspensions of C57BL/6 (C57) and K14.E7 epidermis were analyzed for different CD45^+^ (myeloid) antigen presenting cell subsets. **(A)** Gating strategy to identify LCs, CD11b^+^ and CD103^+^ DCs in C57BL/6 and K14.E7 epidermis. Plots were pre-gated on live CD45^+^ cell singlets. **(B)** Relative proportions of LCs, CD103+ and CD11b+ DC subsets from CD11c^+^ DCs in K14.E7 and C57BL/6 epidermis. **(C)** Relative (as % of CD45^+^ live cells) and absolute number of LCs in the epidermis of two K14.E7 and C57BL/6 mouse ears **(D)** Expression of cell surface markers MHCII, CD86, CD80, CD207 and CD11b on epidermal LCs from C57BL/6 and K14.E7 ear skin. Typical histograms (C57 = black, K14.E7 = white) and pooled median fluorescent intensity (MFI) data (n = 6, two independent determinations) are shown.

In healthy skin, Langerhans cells (LCs) are thought to be the only myeloid cell population in the epidermis and to represent the first line of defence against pathogens invading the skin [12]. We analysed epidermal myeloid cells from K14.E7 transgenic and non-transgenic ear skin and found while LCs represented the main DC population in both groups, K14.E7 mice additionally had significant numbers of CD103^+^ and CD11b^+^ DCs in the epidermis (Fig 1A and 1B), leading to a decrease of LCs in percentage from the overall DC population. However, both the percentage of LCs from live cells and the total number of LCs in ear epidermis were significantly elevated in K14.E7 mice (Fig 1C).

Expression of co-stimulatory molecules and surface markers on epidermal LCs varied between K14.E7 and control mice. Epidermal LCs from K14.E7 mice expressed significantly less surface MHCII molecules, while CD86 and CD80 expression was slightly elevated (Fig 1D). Furthermore, the expression of both CD11b and CD207 (Langerin) in LC was significantly reduced in K14.E7 mouse epidermis.

### En route LCs and other DCs in the dermis of K14.E7 mice

**Fig 2:**
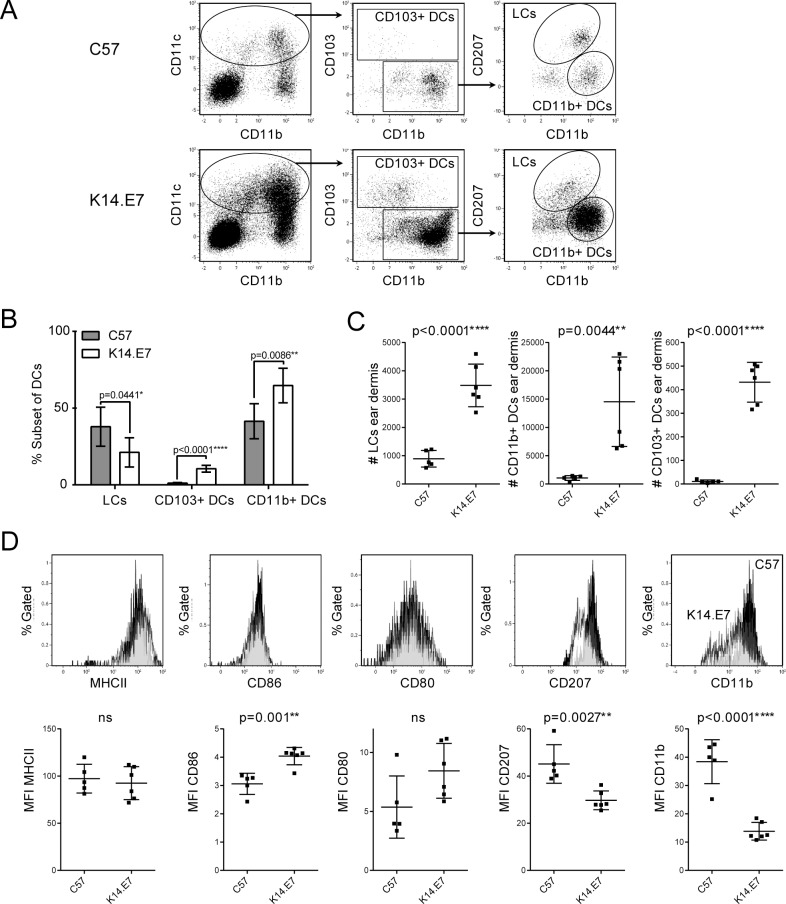
LCs, CD11b^+^ and CD103^+^ dDCs are increased in K14.E7 transgenic mouse dermis. Single cell suspensions of C57BL/6 and K14.E7 dermis were analyzed for different CD45+ (myeloid) antigen presenting cell subsets. **(A)** Gating strategy to identify LCs, CD11b^+^ and CD103^+^ DCs in C57BL/6 and K14.E7 dermis. Plots were pre-gated on live CD45^+^ cell singlets. **(B)** Relative proportions of LCs, CD103+ and CD11b+ DC subsets from CD11c^+^ DCs in K14.E7 and C57BL/6 dermis. **(C)** Absolute number of LCs, CD11b^+^ and CD103^+^ DCs in the dermis of two ears. **(D)** Expression of cell surface markers MHCII, CD86, CD80, CD207 and CD11b on dermal LCs from C57BL/6 and K14.E7 ear skin. Typical histograms (C57 = black, K14.E7 = white) and pooled median fluorescent intensity MFI data (n = 6, two independent determinations) are shown.

In the dermis of healthy skin, the majority of APCs are CD11b^+^ classical dermal DCs (dDCs), with smaller populations of en route migrating LCs and CD103^+^ dDCs. CD11b^+^ dDCs are also the majority APC population in K14.E7 dermis. The absolute numbers of all three DC subsets was significantly increased in K14.E7 dermis (Fig 2C) and there was a relative increase in percentage of CD103^+^ dDCs and a relative decrease in percentage of LCs from the overall DC population (Fig 2A and 2B). The epidermal LC phenotype was only partly maintained by dermal en route LCs, as CD11b and CD207 expression was reduced, but MHCII expression was unaffected (Fig 2D). MHCII expression was also unaffected in CD11b^+^ and CD103^+^ dDCs (data not shown). These data demonstrate that HPV16-E7-induced epithelial hyperplasia in K14.E7 mice is associated with an influx of DC subsets into both epidermis and dermis characterised by reduced expression of CD11b and CD207, and of MHCII specifically on epidermal LCs.

### Epidermal LCs of K14.E7 mice express immune-modulatory cytokines and enzymes

**Fig 3:**
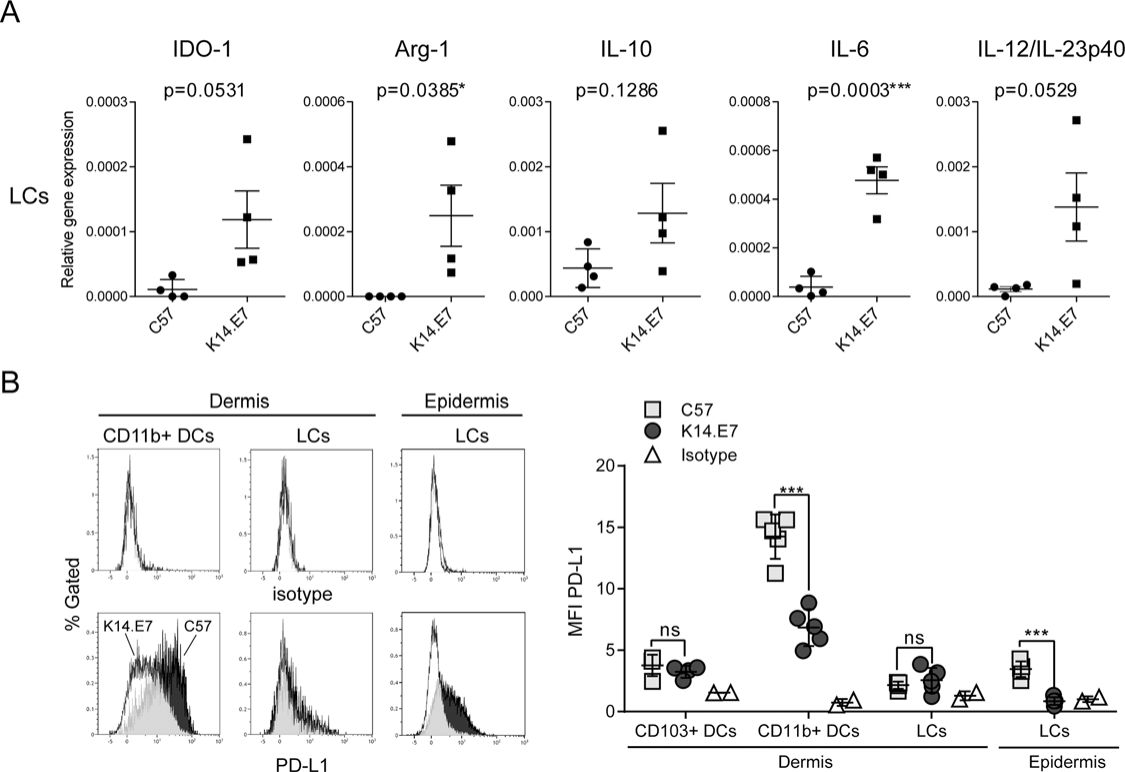
Epidermal LCs of K14.E7 mice express immune-modulatory cytokines and enzymes. **(A)** Single cell suspensions of C57BL/6 and K14.E7 epidermis were prepared. LCs (singlets, live CD45^+^ CD11c^+^ MHCII^+^ CD11b^+^ Epcam^+^ CD103^-^) of K14.E7 and C57BL/6 epidermis were sorted with purity above 96%. Relative gene expression of IDO-1, Arg-1, IL-10, IL-6 and IL12/23p40 messenger RNA was determined by real-time qPCR and normalized against housekeeping gene RPL32. Data are presented as means ±SEM of four independent experiments in which ear epidermis of either 4 C57BL/6 or 2 K14.E7 mice was pooled prior to LC isolation. **(B)** Single cell suspensions of C57BL/6 and K14.E7 dermis and epidermis were analyzed for the expression of PD-L1 on different CD45+ antigen presenting cell subsets. Gating strategy to identify LCs, CD11b^+^ and CD103^+^ DCs in C57BL/6 and K14.E7 skin was performed as shown in Fig 1A and Fig 2A. Expression of cell surface PD-L1 on dermal and epidermal DCs was analyzed. Representative histograms (C57 = black, K14.E7 = white) of CD11b+ DCs, LCs and matching isotype controls, and pooled median fluorescent intensity MFI data (n = 5) are shown.

Reduction of MHCII expression is likely to impact the T cell response primed by DCs. DCs also secrete cytokines and other factors that shape the type of cell-mediated response they initiate, which can be either pro- or anti-inflammatory or result in immune-tolerance. Hence, we selected a range of immune-modulatory factors commonly produced by DCs and known to contribute to a tolerogenic immune microenvironment and tested their expression by epidermal LCs. We isolated epidermal LCs of K14.E7 and non-transgenic mice and analysed the mRNA expression of cytokines IL-6, IL12/IL23p40 and IL-10, and of immune-modulatory enzymes IDO-1 and arginase-1 (Arg-1). We found that K14.E7 epidermal LCs expressed elevated levels of IDO-1, Arg-1, IL-12/23p40 and IL-6 (Fig 3A) suggesting that epidermal LCs have an immune-regulatory function as a result of HPV16-E7 expression in skin. We also analysed the surface expression of programmed death ligand 1 (PD-L1), which is found to be expressed by HPV+ cervical cancers and head and neck cancers [25–27]. Interestingly, the expression of PD-L1 specifically on CD11b+ dermal DCs and epidermal LCs was significantly reduced in K14.E7 skin (Fig 3B), which suggests that immune-suppression in K14.E7 skin is not mediated through PD-L1 on DCs.

### K14.E7 DCs take up antigen in the skin and migrate to draining lymph node

**Fig 4:**
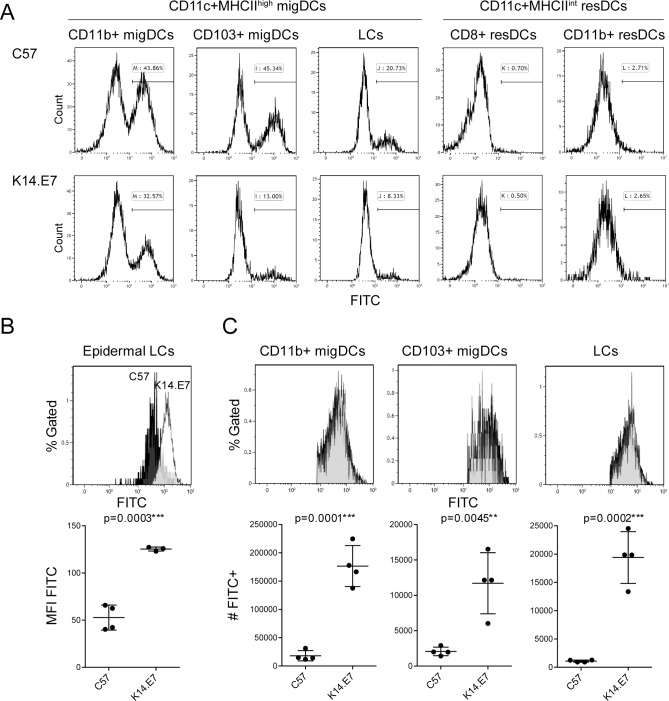
Skin-resident DCs of K14.E7 mice take up antigen and migrate to the draining lymph nodes. C57BL/6 and K14.E7 mice were painted with 100μl of 5mg/ml FITC dissolved in 1:1 acetone:dibutylphalate on shaved flanks and ears. 24 hours later, axillary and inguinal lymph nodes were analyzed by flow cytometry for presence of FITC^+^ DC subsets. **(A)** FITC uptake by DC subsets in lymph nodes pre-gated according to Table 1 and S1 Fig. **(B)** MFI of FITC^+^ LCs in the ear epidermis pre-gated according to Fig 1A. **(C)** MFI and total number of FITC^+^ DCs in lymph node. Shown is one of two independent experiments, n = 4.

APC subsets take up antigen in the tissue, process it for presentation, migrate to draining lymph nodes and mature to express co-stimulatory molecules and cytokines in order to effectively induce a cell-mediated response. To examine this process in more detail in K14.E7 mice, we painted skin of non-transgenic and K14.E7 mice with FITC, and monitored antigen uptake and delivery by APCs to the draining lymph node (Fig 4A). We assessed both migratory DC subsets (migDCs) (LCs, CD11b^+^ migDCs and CD103^+^ migDCs) and LN-resident DC subsets (resDCs) (CD8^+^ resDCs and CD11b^+^ resDCs) according to gating strategies shown in S1 Fig and Table 1. To determine whether there was direct passage of FITC from the skin to the lymph node, we assessed whether LN-resident DCs had taken up FITC, and no such uptake was observed (Fig 4A). Rather, FITC+ve cells in the lymph node had the phenotype of migratory DC subsets, which we infer had transported FITC from skin to LN. FITC median fluorescent intensity (MFI) was significantly higher in the epidermal LCs of K14.E7 mice than in non-transgenic animals, suggesting that K14.E7 epidermal LCs had a higher FITC uptake (Fig 4B). In the draining lymph node, the relative percentage of FITC+ DCs from all DC subsets was decreased in K14.E7 mice (Fig 4A). But of more biological relevance, the FITC MFI in the migratory FITC^+^ DC subsets was comparable in K14.E7 and non-transgenic mice, and an increased absolute number of FITC^+^ migratory DCs was found in the lymph node of K14.E7 mice (Fig 4C). These data suggest that the capacity of APCs in the skin of K14.E7 mice to take up antigen and transport it to the draining lymph nodes is not impaired.

**Table 1:** DC subsets analyzed in lymph nodes and skin and their identification by surface maker stainings.

Organ	DC subset	Markers
Lymph node	CD11b^+^ resDCs	CD11c^+^ MHCII^int^ CD11b^+^ Epcam/CD207^-^ CD8^-^
Lymph node	CD8^+^ resDCs	CD11c^+^ MHCII^int^ CD11b^-^ CD8^+^
Lymph node	CD11b^+^ migDCs	CD11c^+^ MHCII^high^ CD11b^+^ Epcam/CD207^-^ CD103^-^
Lymph node	CD103^+^ migDCs	CD11c^+^ MHCII^high^ CD11b^-^ Epcam/CD207^+^ CD103^+^
Lymph node	LCs	CD11c^+^ MHCII^high^ CD11b^+^ Epcam/CD207^+^ CD103^-^
Skin-Dermis	CD11b^+^ dDCs	CD11c^+^ MHCII^+^ CD11b^+^ Epcam/CD207^-^ CD103^-^
Skin-Dermis	CD103^+^ dDCs	CD11c^+^ MHCII^+^ CD11b^-^ Epcam/CD207^+^ CD103^+^
Skin-Dermis	LCs	CD11c^+^ MHCII^+^ CD11b^+^ Epcam/CD207^+^ CD103^-^
Skin-Epidermis	LCs	CD11c^+^ MHCII^+^ CD11b^+^ Epcam/CD207^+^ CD103^-^

resDCs: resident DCs; migDCs: migratory DCs; dDCs: dermal DCs; LCs: Langerhans cells; +:positive; -:negative; int: intermediate; high: high expression;

### K14.E7 DCs can induce antigen-specific CD8^+^ T cell proliferation in vitro

**Fig 5:**
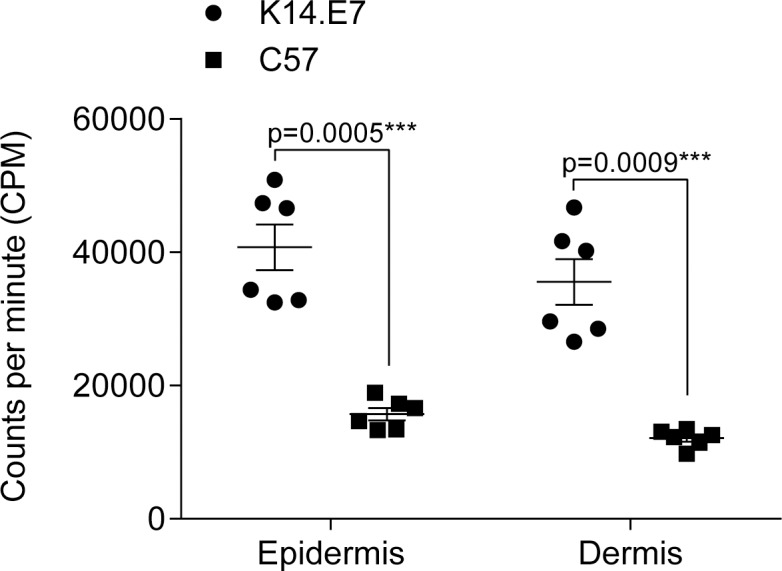
Isolated skin-derived DCs of K14.E7 mice present antigen in vitro, and induce CD8^+^ T cell proliferation. DCs (CD45^+^ CD11c^+^ MHCII^+^) were sorted from ear epidermis and dermis of 10 pooled C57BL/6 and 8 pooled K14.E7 mice and co-cultured together with sorted splenic OT-I CD8^+^ T cells and SIINFEKL at a 1:10 ratio with 5x10^4^ total cells per well. After 72 hours cells were incubated overnight with ^3^H-thymidine and uptake was determined. Shown are triplicates of two pooled independent experiments.

Clearance of a viral skin infection and antigen-specific skin graft rejection each depend on a cytotoxic T cell response (Mattarollo et al., 2011). We therefore analysed the ability of skin-resident DCs from K14.E7 transgenic and non-transgenic skin to activate antigen-specific CD8^+^ T cells. We sorted CD11c^+^MHCII^+^ DCs from epidermis and dermis and co-cultured them with TCR-transgenic OVA-specific OT-I CD8^+^ T cells and SIINFEKL (Fig 5). DCs in healthy skin, in the absence of danger- or pathogen-associated signals, promote tolerance [14]. We observed a limited proliferation of OT-I CD8^+^ T cells when activated by DCs of non-transgenic skin with antigen (Fig 5) that was comparable to control proliferation without antigen (S2 Fig). In contrast, both dermal and epidermal DCs of K14.E7 mice enabled substantially increased T cell proliferation compared to DCs from control animals. We obtained comparable results when we used whole OVA protein (data not shown). Combined these data demonstrates that skin-derived DCs of K14.E7 mice are not impaired in their ability to process and present antigen or to induce antigen-specific T cell proliferation ex vivo. Furthermore, these data suggest that, ex vivo, skin DCs of K14.E7 mice display an activated phenotype compared to non-transgenic control DCs.

### OVA antigen processing in skin-derived K14.E7 DCs is reduced and negatively effects antigen-specific T cell priming in vivo

**Fig 6:**
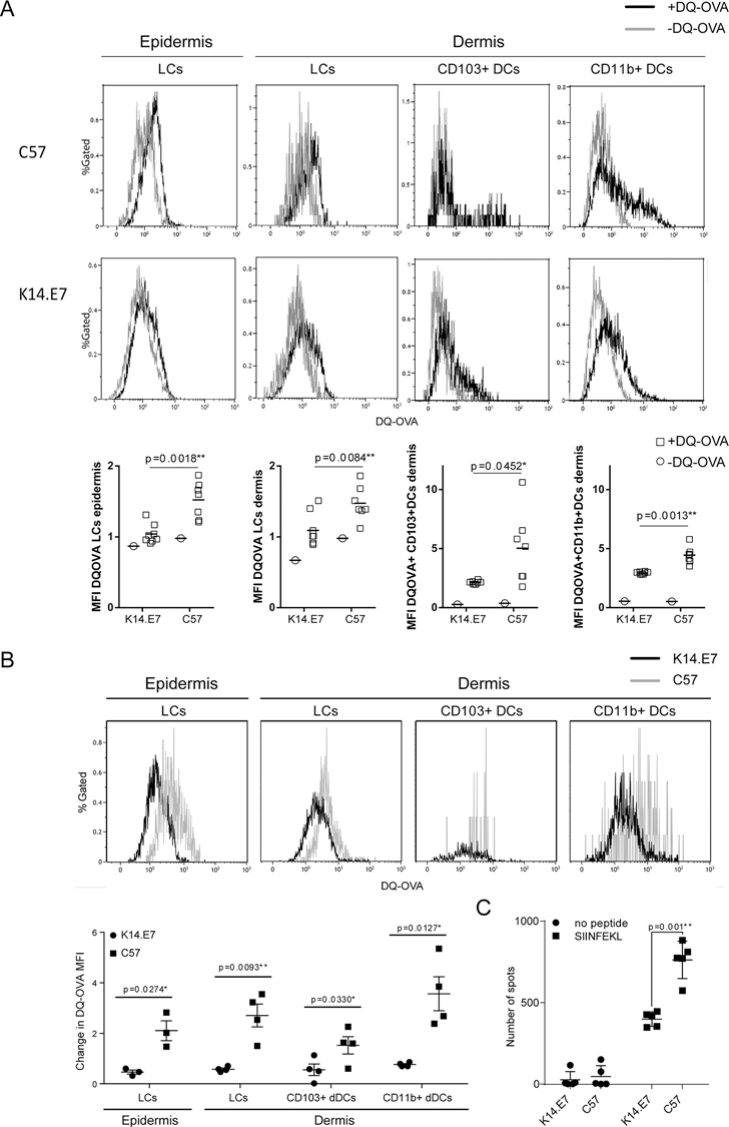
Skin-resident DCs of K14.E7 mice are impaired in their ability to process antigen in vivo. **(A)** Whole dermal and epidermal single cell suspensions of C57BL/6 and K14.E7 ear skin were incubated with 1ug/ml DQ-OVA for 1 hour and analyzed for photolytic degradation by flow cytometry. DC subsets were gated according to Table 1. The green median fluorescent intensity of whole LCs, DQ-OVA+ CD103+ DCs and DQ-OVA+ CD11b+ DCs was compared. **(B)** C57BL/6 and K14.E7 mice were injected intradermally into the ear with 20μl of 1mg/ml labeled DQ-OVA. 24 hours later, single cell suspensions of treated and untreated ear dermis and epidermis were prepared and analyzed by flow cytometry for photolytic degradation of applied DQ-OVA, as recognized by fluorochrome unmasking. Histogram overlays of fluorescence in LCs, CD103^+^ dDCs and CD11b^+^ dDCs determined according to Table 1 in one representative C57BL/6 (grey line) and K14.E7 (black line) mouse ear treated with DQ-OVA. The change in green median fluorescent intensity from untreated to DQ-OVA treated in dermal and epidermal LCs, CD103^+^ dDCs and CD11b^+^ dDCs from K14E7 (●) and C57BL/6 (■) mouse ears (n = 4) was compared. **(C)** C57BL/6 and K14.E7 mice were injected intravenously with OT-I CD8+ T cells and immunized intradermally into the ear pinnae with 20μl of 1mg/ml OVA. One week later, IFNγ secretion by OT-I T cells was analyzed by recalling ear-draining lymph node cells with SIINFEKL antigen in an ELISpot assay. Depicted are spots per 2x10^5^ cells. Each data point represents means of triplicates of one individual animal (n = 5).

In contrast to our observation that APCs in K14.E7 transgenic mouse skin demonstrated enhanced migration and capacity to induce CD8 T cell proliferation *ex vivo*, we have previously observed that K14.E7 transgenic mouse skin provides an immunological microenvironment that is locally suppressive of graft rejection [19]. In an attempt to resolve this paradox, we therefore assessed the capacity of APCs within K14.E7 transgenic skin to process antigen. We first incubated whole skin single cells with ovalbumin conjugated to a dye that exhibits green fluorescence only upon proteolytic degradation, DQ-OVA, and analysed DQ-OVA fluorescence in different DC subsets (Fig 6A). All analysed DC subsets in K14.E7 skin, including LCs, dermal CD11b+ DCs and dermal CD103+ DCs, displayed limitation in DQ-OVA processing compared to control DCs. To verify this finding in vivo, we injected K14.E7 and non-transgenic mice intradermally with DQ-OVA. We separated dermis and epidermis 24 hours after injection and again analysed DQ-OVA fluorescence in all DC subsets. We confirmed that DQ-OVA processing, as assessed by intracellular fluorescence, was reduced in all DC subsets in K14.E7 mice compared to non-transgenic mice (Fig 6B). These data demonstrate that skin-resident DCs of K14.E7 mice, when tested in situ, are relatively impaired in uptake or processing of soluble antigen compared to DCs in non-transgenic skin. To address whether the observed impairment in antigen-processing affects T cell priming in vivo, we immunized K14.E7 and control mice harbouring OT-I CD8+ T cells intradermally with OVA. One week later we assessed the priming capacity of K14.E7 skin-derived DCs by analysing IFNγ secretion by OT-I T cells upon antigen recall. We found that OT-I priming in K14.E7 mice was significantly reduced compared to control mice (Fig 6C). This data supports the hypothesis that skin-derived DCs of K14.E7 mice are functionally impaired.

## Discussion

Here, we have observed significant changes in numbers and markedly altered function of antigen presenting cells in hyperproliferative skin expressing the E7 protein of HPV16 as a transgene in the mouse. Ear skin of the K14.E7 mouse mimics hyperplastic pre-cancerous human HPV infected skin, in that it has a hyperplastic epithelium with steady state expression of E7 in basal epithelial cells without expression of other viral proteins, and thereby can give insight on the mechanisms by which HPV associated premalignant lesions can persist and alter skin immune homeostasis [18, 28]. Grafted K14.E7 ear skin on immune-competent hosts is tolerated despite the expression of HPV16-E7 as a neo-antigen. Immune tolerance is associated with an immune-suppressive local microenvironment within HPV16-E7-expressing skin [19–21, 23, 29]. While some factors involved in this immune suppression have been identified, the exact mechanism by which induced antigen-specific cytotoxic effector T cells are locally impaired in function, is still undefined. K14.E7 skin grafts can be rejected from immune-competent hosts when certain immune suppressors are removed from the skin. Rejection of K14.E7 skin grafts depends on CD8^+^ cytotoxic T cells [28], and immune mediators such as NKT cells, mast cells, IFNγ, IL-17 and IDO play a crucial role in immune suppression [19–21, 23, 30]. That the suppression of cytotoxic effector function is local is demonstrated by parallel grafting of antigen expressing skin, with and without the local suppressive factor, on the same animal results in rejection of only the graft lacking the suppressive factor [19].

As naïve T cells are mainly activated by migratory DCs following epicutaneous infection [15], immune-regulatory mediators likely work by altering APC function. We have previously published that LC homeostasis and activation in the epidermis of K14.E7 skin are altered [22]. In this report, we confirmed the increase and altered activation status of epidermal LCs. We further analysed the immune-modulatory characteristics of LCs in HPV16 E7-expressing epidermis and also analysed other DC subtypes in epidermis and dermis and their ability to migrate, to process antigen and to activate antigen-specific CD8^+^ T cells. This elaborate analysis lead to a series of new observations which the expression of HPV16 E7 has on different APCs in different skin compartments.

Under steady state conditions dermal DCs and epidermal LCs constantly migrate to skin-draining lymph nodes to induce tolerance [14, 31–33]. Inflammation induced by skin injury, infection, malignancy or autoimmune processes enhances the migration of DCs to and from the skin to initiate cell-mediated immune responses [34]. In contrast, HPV persistence has been associated with inhibition of APC adhesion, antigen uptake and migration [35]. Overexpression of E7 has been shown to block endosome acidification, a process crucial to antigen processing and presentation by DCs [36]. HPV^+^ cervical cancer cells were shown to inhibit the differentiation of monocytes to Langerhans cells in vitro [37]. Furthermore E7 down-regulates E-cadherin, which disrupts the adhesion of LCs and keratinocytes [35, 38, 39] and silencing of E7 has been shown to restore E-cadherin expression and LC adhesion [40]. HPV has also been shown to interfere with CCL20 expression in keratinocytes, a chemokine attracting LCs to remain in the epidermis [41]. In our mouse model with HPV16-E7-expressing keratinocytes we found that total numbers of LCs in epidermis and dermis were increased. Epidermal LCs in K14.E7 mice further expressed less MHCII, CD11b and CD207. A reduced MHCII expression on myeloid and monocyte-derived DCs has been observed in blood of women with HPV-related cervical squamous intraepithelial lesions [42], suggesting that DCs have deficiencies in the ability to induce cell-mediated immunity. These K14.E7 skin LCs further expressed an activated phenotype with increased CD80, CD86, and IL-12/IL-23p40, and an immune-regulatory phenotype characterised by increased IDO-1, Arg-1, and IL-6, each of which can be explained to contribute to the immune-suppressive microenvironment found in HPV16-E7-expressing skin. A tolerogenic DC is not necessarily a DC that lacks maturation as it can prime distinct T effector responses such as regulatory T cells through the expression of third activation signals such as IL-10, PD-L1 or IDO. IL-17 produced by CD4^+^ and γδT cells was shown to be immune-suppressive in the HPV16-E7 skin grafting model [20] and both IL-6 and IL-12/IL-23p40 play a role in differentiation of IL-17-producing T cell subsets. Arg-1 has been described as both immune-suppressive and pro-inflammatory factor [43–45]. Further studies will investigate whether the altered mRNA expression levels we describe translate to altered protein expression.

We found that specifically dermal CD11b+ DCs and epidermal LCs in K14.E7 skin expressed significantly less PD-L1 than these DCs subsets in control skin. It has been reported that the main mononuclear cells expressing PD-L1 in HPV+ cervical cancer and head and neck cancers are CD8+ lymphocytes [25]. This further correlated with a poor activation status of CD8+ T cells. We have also observed significantly elevated expression of PD-L1 on CD45+ non-DCs and CD103+ non-DCs (presumably T cells) in K14.E7 skin (data not shown). Our data however indicate that PD-L1 on DCs might not contribute to immune suppression in HPV16 E7-induced hyperplasia.

DCs of K14.E7 skin further showed an increased antigen uptake and migration to skin-draining lymph nodes. Ex vivo, they were competent to induce T cell proliferation when isolated from the skin. All these observations suggest that DCs of K14.E7 skin display a very mature and activated phenotype. However, when they were challenged to process antigen in situ within the skin and prime an antigen-specific T cell response, their ability to do so was decreased, suggesting that DCs in K14.E7 skin are repressed by the specific immune microenvironment.

As CD11b^+^ and CD11b^-^ DCs are functionally specialized for priming CD4^+^ or CD8^+^ T cell responses respectively [46, 47], it will be helpful to understand if and how HPV16 E7 expression affects these subsets. Given the lack of success to date in development of specific immunotherapy for persisting HPV infection and consequent cancers [48], elucidating the mechanisms by which HPV16-E7 modulates different DC subsets in both dermis and epidermis to impair local T cell effector functions may potentially provide new targets for therapeutic immunotherapy.

## Material and Methods

### Mice

C57BL/6 and HPV16-E7-transgenic C57BL/6 mice (K14.E7) were obtained from the Animal Resources Centre (Perth, Australia). All mice were kept under specific pathogen-free conditions at the Biological Research Facilities of Princess Alexandra Hospital and Translational Research Institute and were used at 6–12 weeks of age.

#### Ethics statement

All animal procedures and experiments were performed in compliance with the ethical guidelines of the National Health and Medical Research Council of Australia, with approval from the IMVS Animal Ethics Committee and the University of Queensland Animal Ethics Committee (#290–10 and #367–13).

#### Lymphocyte isolation from lymph nodes

Mice were euthanized in CO_2_ and lymph nodes were removed. Axial and inguinal lymph nodes were cut into small pieces and digested with 0.5 mg/ml collagenase D (Roche, Germany) diluted in PBS 10% FCS for 60 min at 37°C. Lymph nodes were disintegrated by straining though a 70μm cell strainer (BD Pharmingen). Cell counts were performed using Countess Automated Cell Counter (Invitrogen).

#### Lymphocyte isolation from skin

Mice were euthanized in CO_2_ and ears were removed. Ear skin was split with forceps into dorsal and ventral sides and floated epidermis facing down in 1.2 mg/ml Dispase diluted in PBS (Roche, Berlin, Germany) for 60 min at 37°C. The thin epidermal layer was stripped from dermis using forceps. Dermis and epidermis were further processed separately and cut into small pieces followed by digestion with 0.5mg/ml of collagenase D (Roche, Germany) diluted in PBS 10% FCS for 60 min at 37°C. The remaining tissue was disrupted by straining through an 18`G needle and washing through a 70μm cell strainer (BD Pharmingen). Total cell isolation of two ears/ one animal was used for flow cytometry analysis.

#### Reagents and flow cytometry

Anti-mouse monoclonal antibodies (mAbs) to CD16/32 (2.4G2), CD45 (104), CD8 (SK1), CD11c (HL3), CD11b (M1/70), CD207 (929F3.01), CD103 (M290), MHCII (M5/114.15.2), PD-L1 (10F.9G2) and associated isotype controls were purchased from BioLegend (San Diego, CA), eBioscience (San Diego, CA), BD Pharmingen (San Diego, CA) and Dendritics (Kenilworth, USA). For staining of myeloid cells, cells were incubated with mAbs to CD16/32 and live/dead cell fluorescent reactive dye (Invitrogen Molecular Probes, USA) for 20 min at 4°C to block unspecific staining and exclude dead cells. All mAbs were incubated for 20 min at 4°C at predetermined optimal concentrations. For intracellular staining of CD207, cells were fixed and permeabilized using the BD Cytofix/Cytoperm kit according to manufacturer’s instructions (BD Pharmingen). Cells were acquired on a Gallios^TM^ flow cytometer (Beckman Coulter) and analysed using Kaluza software (Beckman Coulter).

#### RNA isolation of epidermal LCs and real-time qPCR

Epidermal single cell suspensions were prepared as described above. Cells were stained and gated for alive-CD45^+^CD11c^+^MHCII^+^CD11b^+^CD326^+^ cells and sorted on a Beckman and Coulter Aria cell sorter (BD Biosciences) with purities above 96%. RNA was isolated with the ISOLATE II RNA microkit (Bioline) according to the quick-start protocol provided by the kit. RNA was reverse transcribed using MuLV Reverse Transcriptase (Applied Biosystems) using a Mastercycler nexus PCR machine (Eppendorf) to produce cDNA. Real-time qPCR was performed using SYBR Premix EX Taq II and ROX reference dye and run on an AB7900HT PCR machine (Applied Biosystems). Sample signals were normalized to the housekeeping gene RPL32 according to the ΔΔCt method: ΔCt = ΔCt_sample_ - ΔCt_reference_ and relative mRNA expression against control sample were calculated. Primers: RPL32: *5’-acaatgtcaaggagctggag-3’*, *5’-ttgggattggtgactctgatg-3’*; IL-6: *5’-tatgaagttcctctctgcaagaga-3’*, *5’-tagggaaggccgtggtt-3’*; IL-12/IL-23p40: *5’-gaccatcactgtcaaagagtttctagat-3’*, *5’-aggaaagtcttgtttttgaaattttttaa-3’*; IDO-1: *5’-tgtccgtaaggtcttgccagg-3’*, *5’-cgaaatgagaacaaaacgtcc-3’*; ARG-1: *5’-aagaatggaagagtcagtgtgg-3’*, *5’-gggagtgttgatgtcagtgtg-3’*; IL-10: *5’-agccgggaagacaataactg-3’*, *5’-ggagtcggttagcagtatgttg-3’*

#### FITC skin paint

Shaved flanks and ears were painted with 100μl of 5mg/ml FITC (Invitrogen) diluted in 1:1 acetone:dibutylphtalate. 24 hours later, mice were euthanized in CO_2_ and ear skin and LNs were removed and processed for flow cytometry as described above.

#### Co-cultures of DCs and OT-I T cells

Single cell suspensions of dermis and epidermis were prepared and stained with surface antibodies as described above. For DC isolation, ear dermis and epidermis cells of either 6 K14.E7 mice or 12 C57BL/6 mice was pooled and alive- CD45^+^CD11c^+^MHCII^+^ cells were sorted. For OT-1 T cell isolation, alive-TCRb^+^CD8^+^ cells were sorted from spleens of OT-1 mice. For sorting, cells were resuspended in complete media (RPMI, 10% FCS, 1% Penicillin and Streptomycin, 1% Glutamine). Cell purities exceeded 90%. DCs and OT-I T cells were plated in 96-round bottom wells at a ratio of 1:10 and 5x10^4^ cells per well. Cells were stimulated with 2μg/ml SIINFEKL peptide for 72 hours.

### Thymidine incorporation assay

72 hours after culture, supernatants were replaced with fresh warm complete media supplemented with 1μCi ^3^H-thymidine overnight or for a maximum of 18 hours. Incorporated radioactivity was determined by Beta Scintillation Counter (Microbeta Trilux, Perkin Elmer, Wellesley, MA).

### DQ-OVA labelling

Single cell suspensions of dermis and epidermis were incubated for 1 hour at 37°C with 1μg/ml DQ-OVA (Molecular Probes, Australia) in complete media, and subsequently antibody stained for flow cytometry. For in vivo DQ-OVA labeing, mice were injected intradermally into one ear with 20μl of 1mg/ml DQ-OVA while the other ear was left untreated. 24 hours later, mice were euthanized, ears were collected and single cell suspensions of dermis and epidermis were prepared as described above for analysis by flow cytometry. We identified LCs, CD11b^+^ dDCs and CD103^+^ dDCs and compared the MFI of green fluorescence (DQ-OVA) between treated and untreated ear.

### Adoptive OT-I transfer, OVA immunizations and IFNγ ELISpot

OT-I CD8+ T cells were isolated from spleens using an EasySep™ Mouse CD8+ T Cell Isolation Kit (StemCell^TM^ Technologies) according to manufacturer’s protocol, and injected intravenously to the tail vain at 1x10^6^ cells per mouse. One day later, mice were immunized intradermally with 20μg of OVA protein to each ear pinnae. One week later, ear-draining lymph nodes were harvested and single cell suspensions were prepared.

For ELISpot assays, 200 000 cells/well were cultured overnight at 37°C with no peptide or 10μg/ml of SIINFEKL. The IFNγ ELISpot procedure was previously described [28]

### Statistics

Two-tailed *t* tests were used to asses differences between groups; *p* values <0.05 were considered significant*, *p* values <0.01**; <0.001*** and <0.0001**** were considered highly significant.

## Supporting Information

S1 Fig Gating strategy to identify DC subsets in skin-draining lymph nodes. Migratory DC subsets (migDCs) were identified as CD11c^+^ MHCII^high^. From these, we discriminated CD11b^+^ migDCs (CD207^-^CD11b^+^) and CD207^+^ DCs. CD207^+^ DCs were further classified into LCs (CD207^+^CD11b^+^) and CD103^+^ DCs (CD207^+^CD11b^-^CD103^+^). Lymph node-resident DC subsets were identified as CD11c^+^MHCII^int^. From these we discriminated CD11b^+^ resDCs and CD8^+^ resDCs. +: positive; -: negative; int: intermediate expression; high: high expression;
(TIF)

S2 Fig Thymidine proliferation assay control data. DCs and CD8^+^ T cells were sorted and cultured within the same assay as described in Fig 5. To determine the unspecific proliferation baseline, we measured ^3^H-thymidine incorporation of T cells only (no DCs, no antigen), T cells and DCs (no antigen), T cells and OVA (no DCs), and T cells and SIINFEKL (no DCs).
(TIF)

## Abbreviations

HPV: human papillomavirus
K14.E7: transgenic expression of oncoprotein
HPV16-E7: under the control of K14 promotor in keratinocytes
cDCs: conventional dendritic cells
MFI: median fluorescent intensity
LCs: Langerhans cells
resDCs: lymph node-resident dendritic cells
migDCs: migratory dendritic cells
dDCs: dermal dendritic cells
MHC: major histocompatibility complex
