# Supplementary figures and images for: Epithelium Expressing the E7 Oncoprotein of HPV16 Attracts Immune-Modulatory Dendritic Cells to the Skin and Suppresses Their Antigen-Processing Capacity
Source: PLoS One. 2016 Mar 31;11(3):e0152886. doi: 10.1371/journal.pone.0152886 (PMC4816461; doi:10.1371/journal.pone.0152886)

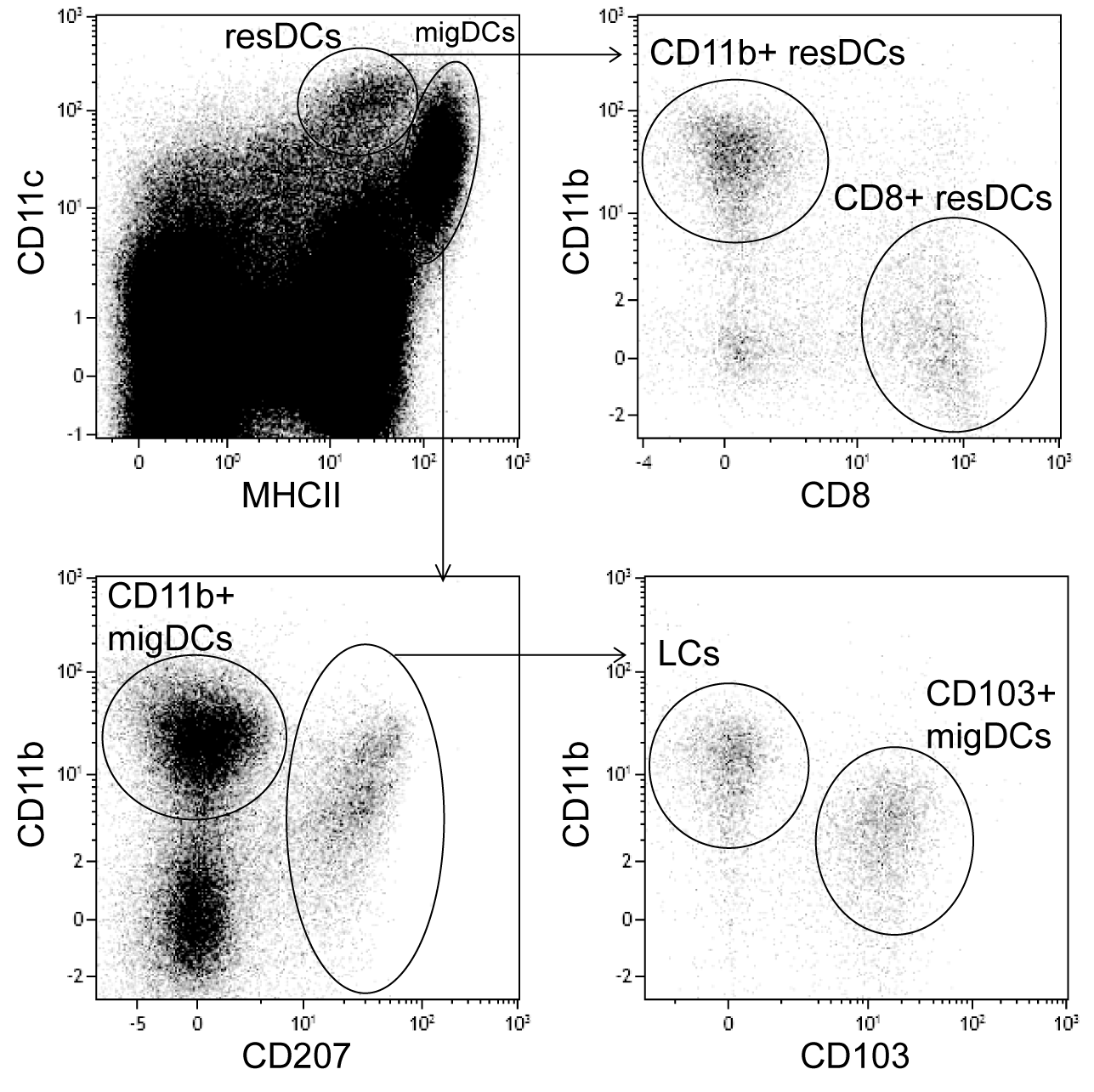

Supplement: S1 Fig — Migratory DC subsets (migDCs) were identified as CD11c+ MHCIIhigh. From these, we discriminated CD11b+ migDCs (CD207-CD11b+) and CD207+ DCs. CD207+ DCs were further classified into LCs (CD207+CD11b+) and CD103+ DCs (CD207+CD11b-CD103+). Lymph node-resident DC subsets were identified as CD11c+MHCIIint. From these we discriminated CD11b+ resDCs and CD8+ resDCs. +: positive; -: negative; int: intermediate expression; high: high expression; (TIF) [file pone.0152886.s001.tif]

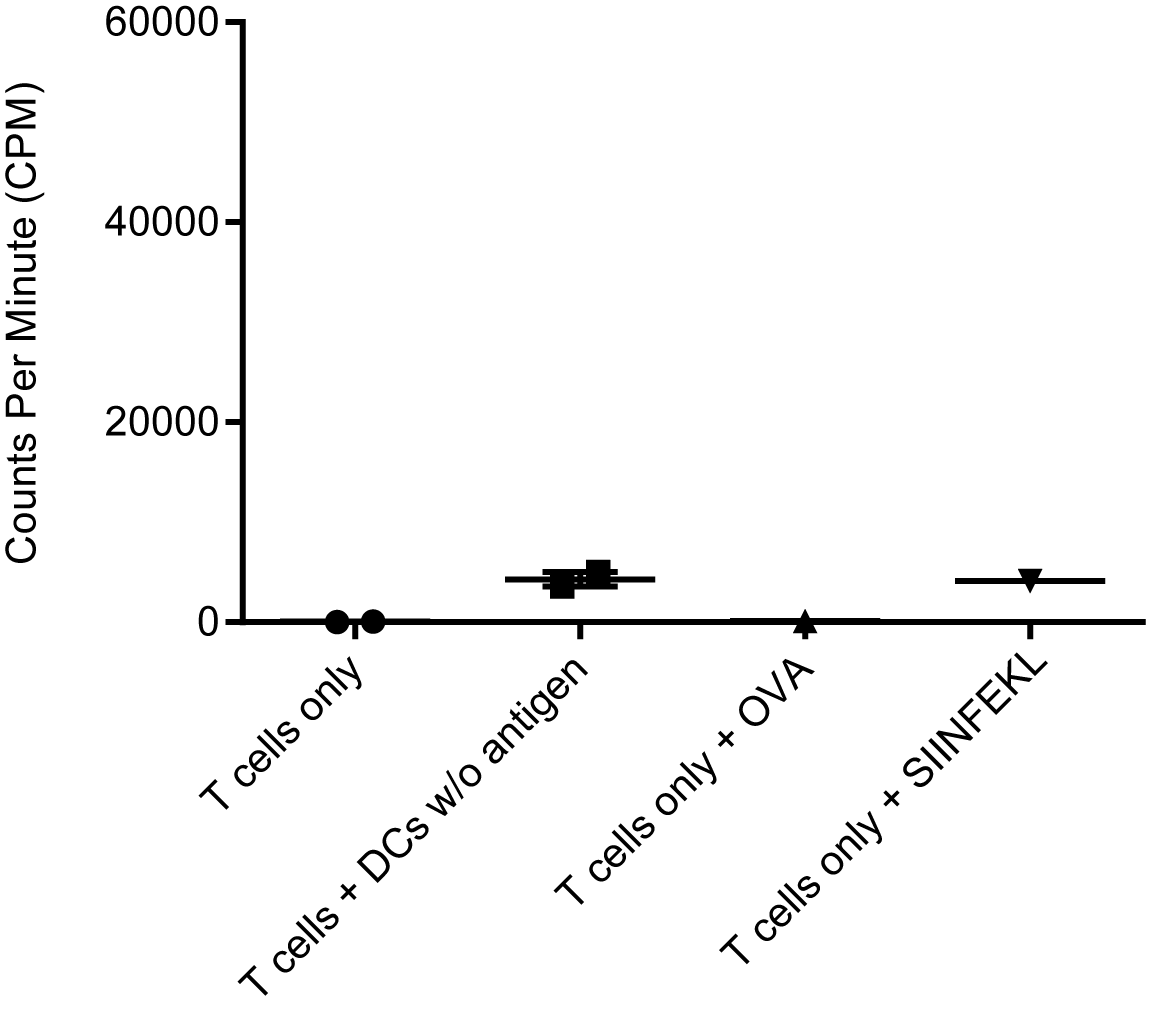

Supplement: S2 Fig — DCs and CD8+ T cells were sorted and cultured within the same assay as described in Fig 5. To determine the unspecific proliferation baseline, we measured 3H-thymidine incorporation of T cells only (no DCs, no antigen), T cells and DCs (no antigen), T cells and OVA (no DCs), and T cells and SIINFEKL (no DCs). (TIF) [file pone.0152886.s002.tif]
